# CAN STATURE, ABDOMINAL PERIMETER AND BMI INDEX PREDICT POSSIBLE CARDIOMETABOLIC RISKS IN FUTURE OBESITY?

**DOI:** 10.1590/0102-672020200002e1529

**Published:** 2020-11-20

**Authors:** Ricardo Wallace das Chagas LUCAS, Paulo Afonso Nunes NASSIF, Fernando Issamu TABUSHI, Denise Serpa Bopp NASSIF, Bruno Luiz ARIEDE, Jose BRITES-NETO, Osvaldo MALAFAIA

**Affiliations:** 1 Postgraduate Program in Principles of Surgery, Mackenzie Evangelical Faculty of Paraná/Medical Research Institute, Curitiba, PR, Brazil; 2 Paulo Nassif Institute, Curitiba, PR, Brazil; 3 Bariatric and Metabolic Surgery Service, University Evangelical Mackenzie Hospital, Curitiba, PR, Brazil

**Keywords:** Obesity, Obesity, abdominal, Metabolic syndrome, Obesidade, Obesidade abdominal, Síndrome metabólica

## Abstract

**Background:**

Obesity changes the anatomy of the patient. In addition to the aesthetic change, the high percentage of fat determines evident functional changes. Anthropometric normality in measuring abdominal circumference and height can serve as a basis for measuring cardiometabolic risks of obesity.

**Aim:**

To verify if it is possible to determine parameters of normality between waist and height in people with normal BMI and fat percentages, to serve as a basis for assessing risks for obesity comorbidities.

**Methods:**

A sample of 454 individuals with BMI and percentages of fat considered within the normal range was extracted. It was divided into age groups for both men and women between 18 and 25; 26 to 35; 36 to 45; 46 to 55; 56 to 65. A total of 249 men and 205 women were included.

**Results:**

Regarding the percentage of height as a measure of the abdominal perimeter, the total female sample had an average of 44.2±1.1% and the male 45.3%+1.5. For women, this percentage determined the equation of the waist-height ratio represented by X=(age+217) / 5.875, and for men X=(age+190.89) / 5.2222. “X” represents the percentage of the height measurement so that the individual falls into the category of adequate percentage of fat and BMI.

**Conclusion:**

Between the stature of adult men and women with normal fat percentage and BMI, there is a common numerical relationship, with is on average 44% for women and 45% for men.

## RESUMO

**Racional:**

A obesidade modifica a anatomia de seu portador. Além da mudança estética o alto percentual de gordura determina evidentes alterações funcionais. A normalidade antropométrica na mensuração do perímetro abdominal e estatura pode servir de base na mensuração dos riscos cardiometabólicos da obesidade.

**Objetivo:**

Verificar se é possível determinar parâmetros de normalidade entre cintura e estatura em pessoas que apresentem IMC e percentuais de gordura normais, para servir de base na avaliação de riscos para co-morbidades da obesidade.

**Métodos:**

Foi extraída amostra de 454 indivíduos com IMC e percentuais de gordura considerados dentro da normalidade. Ela foi dividida em faixas de idade tanto para homens como para mulheres entre 18 a 25; 26 a 35; 36 a 45; 46 a 55; 56 a 65. Totalizou 249 homens e 205 mulheres.

**Resultados:**

Em relação ao percentual da estatura como medida do perímetro abdominal, a amostra total feminina apresentou média de 44,2±1,1% e a masculina 45,3%+1,5. Para as mulheres este percentual determinou a equação da relação cintura-estatura representado por X=(idade+217)/5,875, e para homens X= (idade+190,89) /5,2222. “X” representa o percentual da medida da estatura para que o indivíduo se enquadre na categoria de adequados percentual de gordura e IMC.

**Conclusão:**

Entre a estatura de homens e mulheres adultos possuidores de percentuais de gordura e IMC normais, existe relação numérica comum, sendo em média 44% para mulheres e 45% para homens.

## INTRODUCTION

Obesity has become a worldwide epidemic^7^ ^,^ ^27^. Although there are indications that it is metabolically healthy, there is consensus on its harm and various risks present in obese individuals who have visceral fat deposition^5^ ^,^ ^20^ ^,^ ^23^ ^,^ ^28^. The greater the volume and the percentage of body fat with the increase in body mass index (BMI), there is also deposition in visceral fat^3^. However, the methodological modalities used to measure total body fat are not directly related to visceral fat, which can be detected by ultrasound, tomography and magnetic resonance.

Despite not having high accuracy, anthropometric measurements have been the most used to assess visceral fat. Among them have been those that analyze the perimeters. The use of the waist/hip ratio and the abdominal waist has shown adequate correlations with visceral fat, when estimated by tomography, and adequately predict cardiometabolic risk^7^.

Regarding the measurement of the abdominal perimeter (waist), the adequacy of the cutoff point established at 102 cm for men and 88 cm for women^12^ is questioned for populations of different ethnicities. Some studies with lower levels - 94 cm for men and 80 cm for women - have been considered more appropriate^6^ ^,^ ^10^ ^,^ ^18^ ^,^ ^20^ ^,^ ^28^. It is still mentioned that different ethnicities have different somatotypes, and consequently different fat distributions, so that cut-off values ​​predictive of risk in a given population may not be valid for others. The use of height, as it is relatively immutable after adulthood, has served as a basis for the composition of measures more applicable to different populations^21^.

In this sense, an adult individual with normal body composition, regardless of gender and age group, presents body measurements with numerical values ​​related to height.

Thus, the aim of this study was to study the percentage waist-height ratio in patients with normal fat percentages, and to format a table (Table 3) relating it to age, in order to propose parameters for the assessment of cardiometabolic risks in future obesity.

## METHODS

This prospective descriptive study correlated data collected from the Bariatric and Metabolic Surgery Service of Hospital Universitário Evangélico Mackenzie in Curitiba, PR, Brazil. The sample initially had 1,717 individuals. The data of interest for the study were: height, total body mass (weight), the percentage of fat and the smallest abdominal circumference. The variables were collected as follows: height using a portable stadiometer with a 1 mm metric scale; total body mass by a calibrated portable digital scale with a capacity of 150 kg and a margin of 100 g; abdominal perimeter, with an inextensible measuring tape and 0.1 cm margin; fat percentage, by Omron bipolar bioimpedance equipment, model HBF-306.

**FIGURE 1:**
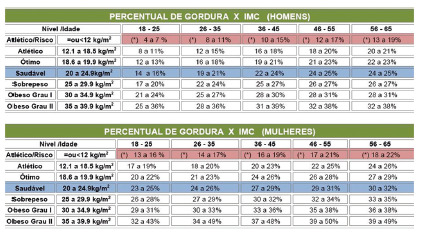
Pollock & Wilmore (1993) table showing BMI vs. % of fat by age group and gender in relation to the state of body complexion

With body composition considered adequate within the initial sample, 454 adult individuals of both genders were extracted in the age group between 18 and 65 years old and who had a normal percentage of fat and BMI (Pollock and Wilmore, 1993). They were divided into groups by age groups, namely: 18 to 25 (n=165 ♂ 81♀); 26 to 35 (n=53♂80♀); 36 to 45 (n=15♂25♀); 46 to 55 (n=13♂ 13♀); 56 to 65 (n=3♂ 6♀, Figure 1). There were 249 men and 205 women.

### Statistical analysis

For data treatment, the Excel statistical program was used. Statistical procedures were carried out following age, height, abdominal circumference, fat percentage and waist-to-height ratio. Pearson’s correlation coefficient (r), mean and standard deviation of all variables were used, and the 95% confidence interval for mean abdominal circumference. The waist-to-height ratios of the population were transcribed in percentages, and the averages of the abdominal perimeter were determined.

## RESULTS

Separating the genders, the standard deviation of the mean of each age group was calculated. Adding all of them, the female sample had an average of 44.2+1.1% in height.

**TABLE 1:** Mean and standard deviation of each stratum of female age group by analyzed parameter (n=205)

Age range	<Abdominal perimeter	Height	Percentual relation	BMI	%Fat
18 a 25	70	164.4	42.6	20.4	19.6
26 a 35	71.7	163.8	43.8	20.9	21.8
36 a 45	73.3	163.2	44.9	21.9	25.7
46 a 55	71.2	161.2	44.1	21.5	25.8
56 a 65	71.7	157.8	45.4	21.8	31.3
Mean	71.6	162.1	44.2	21.3	24.8
SD	1.2	2.7	1.1	0.6	4.5

The male sample had an average height of 45.3%.

**TABLE 2:** Mean and standard deviation of each stratum of male age group per analyzed parameter (n=249)

Age range	<Abdominal perimeter	Height	Percentual relation	BMI	% Fat
18 a 25	75.7	174.9	43.3	21.7	10.3
26 a 35	77.7	175.8	44.2	22	12.6
36 a 45	80.8	176.3	45.8	23	18
46 a 55	79.9	173.6	46.1	22.9	20.1
56 a 65	83.3	176.7	47.1	21.6	20
Mean	79.5	175.2	45.3	22.2	16.2
SD	2,9	1,2	1,5	0,7	4,5

Taking into account the interval between the smallest and the largest range of each gender, a percentage range was found that varied from 39.5% to 47.9% for women and 40.4% to 49.1% for men.

When using Pearson’s correlation between abdominal perimeters and the fat percentage of the female sample, a strong positive correlation was found: R²=0.8559.

**FIGURE 2:**
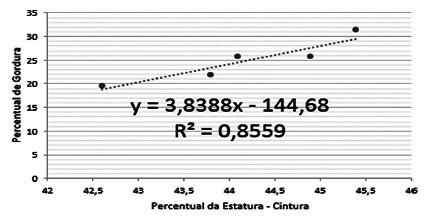
Correlation between abdominal circumference and fat percentage in women

There was also a strong positive correlation (R²=0.9406) when using the Pearson correlation method between the abdominal perimeters analyzed and the fat percentage of the male sample.

**FIGURE 3:**
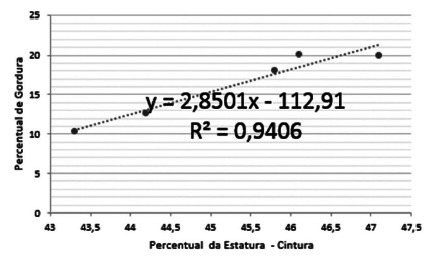
Correlation between abdominal circumference and fat percentage in men

The sample presented nominally for the values of waist in percentage of the height, interval between 40.4% to 49.1% for men and 39.5% to 47.9% for women. Data divided into age groups are shown in Table 3.

**TABLE 3:** Stratum of normal waist-height in relation to gender and age groups in people with normal BMI

Age range	Percentual relation	
	Women	Men
18 a 25	42.6	43.3
26 a 35	43.8	44.2
36 a 45	44.9	45.8
46 a 55	44.1	46.1
56 a 65	45.4	47.1

## DISCUSSION

The focus on the correlation between cardiometabolic disorders present in certain populations and anthropometric or body composition signs, characterizes the waist-height advantage over other anthropometric indices. The waist-to-height ratio is a good discriminator of abdominal obesity related to cardiometabolic risk factors^3^ ^,^ ^5^ ^,^ ^13^ ^,^ ^19^ ^,^ ^22^.

In a study carried out with a sample of 55,563 adults of both genders in Taiwan, with the objective of identifying the cutoff points of the waist-to-height ratio to discriminate at least one cardiovascular risk factor (diabetes, hypertension or dyslipidemia), values ​​of 0.48 were found and 0.45 for men and women, respectively, that is, 48% and 45% of height for men and women. Still in Taiwan, using 38,556 subjects of both genders as a sample, there was a strong association between waist-to-height ratio with arterial hypertension, glucose intolerance, diabetes and dyslipidemia^24^.

Analyzing specifically the results of this research, there was a tendency in the division by strata of age group to increase the percentage of fat with age, even with the individual within the normal BMI range, as shown in Figure 1.

Taking into account that the cardiometabolic risk is higher in older individuals compared to younger ones, there is a correlation with the increase in the measurement of the abdominal perimeter and the percentage of fat found in the research, even in individuals with adequate body composition^3^.

When applying Pearson’s regression equation to the columns referenced by the fat percentage and waist circumference, a strong positive correlation was found for both men and women. This finding corroborates the finding of the waist-to-height ratio when looking for factors associated with central obesity in adults on a population basis^22^.

The stratum of all age groups of men was consistent with the research by Lucas et al.^20^ who, by separating men between 18 and 25 years old, demonstrated that the 43% height range was adequate with a very strong positive correlation (Pearson r=0.778) between the fat percentage and the abdominal perimeter. This data also coincides with the assertion that the higher the percentage of fat, the greater the waist circumference^3^ ^,^ ^13^ ^,^ ^14^. Likewise, it demonstrated a concern for the division of the waist-to-height ratio by strata, in view of the physiological change in body composition with changes in age groups. The loss of lean mass with increasing age may be related to the decrease in its percentage, but it can also increase the fat mass and consequently the fat percentage. The correlation of this physiological finding cannot be exempted from the analysis of the level of physical activity of the studied population, so that anthropometric measures can make sense^21^.

The advantage for the male sample over the correlation between the percentage of fat and the abdominal perimeter is in line with what refers to another anthropometric method of perimetric relationship, that is, using the waist-hip ratio. This reason for women acquires lower values ​​than for men due to the gender trait, which allows the percentage of fat for women - even the essential one - to be higher than for men^8^. Thus, the finding of an unfavorable correlation for women is understandable, even though Pearson’s correlation is strong.

## CONCLUSION

It was possible to determine parameters of normality between waist and height in people with BMI and percentages of normal fat, creating indices (Table 3) for different age groups to assist in the clinical evaluation regarding the risks of comorbidities promoted by obesity.
